# Targeted Thrombolysis with Magnetic Nanotherapeutics: A Translational Assessment

**DOI:** 10.3390/pharmaceutics16050596

**Published:** 2024-04-27

**Authors:** Ming-Lu Lin, Siao-Yun Wu, Jyh-Ping Chen, Yi-Ching Lu, Shih-Ming Jung, Shiaw-Pyng Wey, Tony Wu, Yunn-Hwa Ma

**Affiliations:** 1 Department of Physiology & Pharmacology, College of Medicine, Chang Gung University, Guishan, Taoyuan 33302, Taiwan; 2 Department of Chemical and Materials Engineering, College of Engineering, Chang Gung University, Guishan, Taoyuan 33302, Taiwan; 3 Department of Pathology, Chang Gung Memorial Hospital, Guishan, Taoyuan 33305, Taiwan; ming22@adm.cgmh.org.tw; 4 Department of Medical Imaging and Radiological Sciences, Chang Gung University, Guishan, Taoyuan 33302, Taiwan; spwey@mail.cgu.edu.tw; 5 Department of Neurology, Chang Gung Memorial Hospital, Guishan, Taoyuan 33305, Taiwan; 6 Department of Medical Imaging and Intervention, Chang Gung Memorial Hospital, Guishan, Taoyuan 33305, Taiwan

**Keywords:** targeted thrombolysis, magnetic nanoparticle, recombinant tissue-type plasminogen activator

## Abstract

Plasminogen activators, such as recombinant tissue-type plasminogen activators (rtPAs), while effective in treating thromboembolic diseases, often induce hemorrhagic complications due to non-specific enzyme activities in the systemic circulation. This study evaluated the targeting efficiency, efficacy, biodistribution, and potential toxicity of a rtPA covalently attached to chitosan-coated magnetic nanoparticles (chitosan-MNP-rtPA). The thrombolytic activity of a chitosan-MNP-rtPA was preserved by protection from an endogenous plasminogen activator inhibitor-1 (PAI-1) in whole blood and after circulation in vivo, as examined by thromboelastometry. Single-photon emission computed tomography (SPECT) demonstrated real-time retention of a ^99m^Tc-MNP-rtPA induced by magnet application in a rat embolic model; an 80% reduction in rtPA dosage for a chitosan-MNP-rtPA with magnetic guidance was shown to restore blood flow. After treatment, iron deposition was observed in the reticuloendothelial systems, with portal edema and neutrophil infiltration in the liver at a ten-fold higher dose but not the regular dose. Nevertheless, no liver or renal toxicity was observed at this higher dose. In conclusion, the liver may still be the major deposit site of rtPA nanocomposites after targeted delivery; chitosan-coated MNPs are potentially amenable to target therapeutics with parenteral administration.

## 1. Introduction

In the treatment of thromboembolism, plasminogen activators, such as recombinant tissue-type plasminogen activators (rtPAs, or alteplase), remain the gold standard for acute thrombolytic therapy [1]. However, the impermeability of occlusive clots, inactivation by an endogenous plasminogen activator-1 (PAI-1) [2], and rapid in vivo clearance of a rtPA [3] lead to necessary administration at large doses. Intravenous administration of a rtPA at a pharmacological dose within 4.5 h of symptom onset improves the clinical outcome of ischemic stroke patients [4]; however, intracerebral hemorrhage remains the most common and devastating complication [5]. To overcome these problems and enhance therapeutic efficacy, targeted delivery of a rtPA to the specific site has been investigated [6,7], where magnetic targeting of nanoparticle drug carriers appears to be a reproducible and effective approach.

A magnetic nanoparticle (MNP) is composed of a superparamagnetic iron-oxide core that is crucial for a variety of theranostic applications [8], including magnetic targeting [9]. MNPs can be magnetically guided to and retained at the designated site by overcoming the hemodynamic force in the blood vessel [6]. In vivo studies demonstrated that MNPs primarily accumulated in the liver and spleen despite the use of various coatings [6,10,11].

Pharmacological efficacy has been demonstrated with the rtPA immobilized to various polymer-coated MNPs or magnetoliposomes containing an encapsulated rtPA [6]. With a rat embolic model, MNPs can be guided to the desired site using a mobile NdFeB magnet [12] but not with a static magnet. This suggests that dis-aggregation of the magnetic nanocomposites is paramount for the induction of thrombolysis after magnetic guiding. Since chitosan-based nanoparticles have been shown to possess a relatively long circulation time with low liver uptake [13], a chitosan-coated MNP-rtPA (chitosan-MNP-rtPA) was prepared and characterized [14]. Preliminary studies demonstrated the potential of a chitosan-MNP-rtPA with magnetic guidance in a rat embolic model. Although the biodistribution and toxicity of MNPs with various coatings have been extensively studied [15], there is little or no information regarding how rtPA conjugation may alter the biodistribution or toxicity of a nanocomposite in vivo.

This study evaluated the pharmacological efficacy, biodistribution, and biocompatibility of an immobilized rtPA on a chitosan-MNP-rtPA in vitro and in vivo. Our results demonstrate that with magnetic guiding, rtPA-conjugated magnetic nanocarriers may follow a targeted distribution pattern with only 20% of the dose required to achieve the efficacy compared to a non-targeted system but exhibit final biodistribution pattern similar to that of the nanocarriers.

## 2. Materials and Methods

### 2.1. Materials

The recombinant tissue-type plasminogen activator (rtPA, Actilyse^®^) was purchased from Boehringer Ingelheim (Mannheim, Germany). Inactin^®^ (thiobutabarbital sodium), heparin, penicillin G, and stannous chloride dehydrate were purchased from Sigma (St. Louis, MO, USA). Fe (II) chloride tetrahydrate (≥99%), Fe (III) chloride hexahydrate (≥97%), and α-Naphthylisothiocyanate were purchased from Acros Organics (Morris Plains, NJ, USA). Isoflurane (FORAND^®^) was purchased from Aesica Queenborough Limited (Queenborough, UK). The recombinant human plasminogen activator inhibitor-1 was purchased from Calbiochem (La Jolla, CA, USA). The chitosan polymer (150 kDa) with a degree of deacetylation of 95% was obtained from Fluka (Buchs, Switzerland). The chromogenic substrate, S-2288^TM^, was obtained from Chromogenix (Milano, Italy).

### 2.2. Synthesis of MNP-rtPA

The synthesis of the chitosan-MNP-rtPA was performed as described previously [14]. Briefly, the chitosan-MNP was prepared by the chemical coprecipitation of ferric and ferrous salts in an alkaline medium, followed by coating the MNP with chitosan (4:1; 153 nm in size and zeta potential +23 mV). The recombinant tPA was covalently immobilized to MNP by using a glutaraldehyde method, with a loading efficiency of 95%.

### 2.3. Chromogenic Enzyme Activity Assay

The activity retention of the bound rtPA was determined as the percentage of specific activity of free rtPAs. In the current study, the amount of the active rtPA immobilized on the MNP averaged 100.4 ± 3.3 μg/mg MNP (*n* = 9). In some experiments, the rtPA and MNP-rtPA were preincubated with PAI-1 in molar ratios ranging from 4:1 to 1:1 for 30 min at room temperature, followed by incubation with S-2288TM. The activity retention after incubation with inhibitors was calculated as the percent inhibition of amidolysis = (control − experiment)/control × 100%. The Lineweaver–Burk plot was also used to calculate the inhibition constant (Ki) of PAI-1 at various concentrations. This was accomplished by constructing a double-reciprocal plot, where the inverse of the initial velocity (1/V0) was plotted against the inverse of the substrate concentration (1/[S]).

### 2.4. Thromboelastometry

To assess the process of thrombolysis induced by the rtPA or MNP-rtPA in whole blood, a 4-channel thromboelastometric analyzer (ROTEM^®^, Pentapharm GmbH, Munich, Germany) was used [6]. All measurements were performed according to the manufacturer’s instructions. In brief, citrated rat blood was obtained by cardiac puncture and kept at room temperature for 30 min to 7 h before use. Drugs with CaCl_2_ (12 mM) were placed in a pre-warmed cup before adding 280 μL of citrated blood, followed by a 2-hr observation period. Re-calcification with CaCl_2_ triggered clot formation and gradually increased the elasticity and firmness of the thrombus. The parameters used for analysis as defined by the manufacturer were as follows: (a) clotting time (CT, in second) is the time from the start of the analysis until 2-mm amplitude in firmness recording was achieved and illustrated as a green color in the thromboelastogram; (b) clot formation time (CFT, in second) is the time between the increase in the amplitude of firmness from 2 to 20 mm with tracing in pink in the thromboelastogram; (c) maximal clot firmness (in millimeter) is the maximal firmness detected in the thromboelastogram; and (d) lysis index (LI) is the ratio between maximal clot firmness and amplitude at the indicated time. In some experiments, thromboelastometry was performed with citrated blood (0.4 mL) withdrawn via a jugular vein catheter in anesthetized rats 1, 2, and 5 min after free vs. immobilized rtPA (0.2 mg/kg) administration. More details are available in Supplementary Materials.

### 2.5. Rat Embolic Model

Experiments were performed in a rat embolic model established previously [12,16]. Briefly, 9-week-old Sprague Dawley (SD) rats (363 ± 5 g, *n* = 25; BioLASCO, Taipei, Taiwan) were anesthetized with Inactin (100 mg/kg), followed by tracheostomy and cannulation of the carotid artery for blood pressure measurement. The right iliac artery was cannulated with a catheter for injection of the clot, which was lodged in the left iliac artery. The MNP or MNP-rtPA was injected and guided with an NdFeB magnet (4.9 kG) in a reciprocating motion from aortic bifurcation to the hind limb for one hour. Aortic/iliac flow measurement and tissue perfusion were measured with ultrasonic flowmetry (T206, Transonic Systems; Ithaca, NY, USA) and a laser Doppler perfusion imager (MoorFLPI; Moor Instruments, Devon, UK), respectively. Half reperfusion time was defined as the time required to restore iliac blood flow to 50% of the basal level after drug administration. At the end of the experiment, blood samples were collected by cardiac puncture for hematology analysis by a cell analyzer (HEMAVET^®^ 950LV; Drew Scientific, Cumbria, UK).

### 2.6. Chronic Implantation of the Catheter

Catheterization of the jugular vein was performed for blood sampling as previously described [17]. Briefly, anesthetized SD rats were cannulated with a catheter composed of a 4-cm PE 50 tubing (I.D. 0.58 mm × O.D. 0.97 mm, Clay Adams^®^, Sparks, MD, USA) connected to a 2-cm silicone tubing (I.D. 0.8 mm. × O.D. 1.7 mm, A-M Systems, Carlsborg, WA, USA) filled with heparinized saline (500 unit/mL), with the tip of the silicone tubing reaching the right atrium. An incision of approximately 1 cm was made between the scapulae; a trocar was used to guide the catheter from the neck to the back subcutaneously. The wounds were closed with 3-odd silk sutures. After the surgery, a single intramuscular injection of 50,000 units of penicillin G was administered to prevent infection.

### 2.7. Biodistribution

MNP-rtPA biodistribution was studied in SD rats under magnetic guiding by single-photon emission computed tomography (SPECT)/computed tomography (CT) (NanoSPECT/CT; Bioscan Inc., Washington DC, USA) with a ^99m^Tc-pertechnetate radiolabeled MNP-rtPA. The scintigraphic images were quantitatively analyzed and normalized to steady mean counts of whole-body activity to evaluate the distribution of the ^99m^Tc-MNP-rtPA in vivo. To prepare the ^99m^Tc-MNP-rtPA, 740 MBq ^99m^Tc-sodium pertechnetate (in 0.5 mL of normal saline) was added to the MNP-rtPA solution (10 mg/mL MNP with various concentrations of the rtPA). After adding stannous chloride dehydrate solution (4.5 mg/mL in 0.1 N HCl), the reaction mixture was vortexed for 30 s. and incubated at room temperature for 20 min. After precipitation of the MNP on a magnet, the supernatant containing free ^99m^Tc-pertechnetates was removed. The recovered MNP was washed twice with distilled water to remove residual free ^99m^Tc-pertechnetates and resuspended in distilled water. The radiochemical purity of the ^99m^Tc-MNP-rtPA (>95%) was determined with a radio-thin-layer chromatography (radio TLC) method using an ITLC-SG strip (Gelman Sciences, Inc., Ann Arbor, MI, USA) developed with acetone. The radio chromatogram was analyzed with a radio TLC scanner (AR-2000; Bioscan Inc.). The rat embolic model was prepared under anesthesia as described above. After embolism was induced, 5 mCi ^99m^Tc-MNP-rtPA was injected into the left iliac artery via a catheter in the right iliac artery for a 5-min-period. The magnetic guidance was implemented by an NdFeB magnet in a back-and-forth motion for 30 min. The scintigraphic imaging was performed with 10 s interval dynamic planar images beginning 5 min before drug administration and continuing for 1 h after.

### 2.8. In Vivo Toxicology Assessment

SD rats were anesthetized with isoflurane. Phosphate buffered saline (PBS), α-Naphthylisothiocyanate (ANIT, a well-known hepatotoxicity inducer [18] dissolved in corn oil; 60 mg/kg, i.e., as positive control), or MNP-rtPA (0.15 or 1.5 mg/kg with MNP doses of 2 vs. 20 mg/kg) was injected through the jugular vein. Blood was collected 2 h before and 1 to 28 days after the administration of the MNP-rtPA. For blood sampling, 300 μL of blood was collected into the EDTA.K3-containing tube for the hematologic analysis (HEMAVET^®^ 950LV; Drew Scientific). Then, 600 μL of blood was incubated at room temperature for 20–30 min followed by centrifugation at 3000× *g* for 10 min. The serum thus obtained was stored at −20 °C until the biochemical analysis (Fuji Dri-Chem Clinical Chemistry Analyzer FDC-3500, Sysmex, Hyogo, Japan). The catheter was filled with heparinized saline between blood samplings. At the end of the experiments, the organs of the rats were collected for histology analysis.

### 2.9. Histology Analysis

The rats were anesthetized for the insertion of an aortic catheter from the left ventricle and systemic perfusion of formaldehyde. A small incision was made in the right auricle to allow efflux of the perfusate. Systemic circulation was flushed with 0.9% NaCl at 10 mL/min for 10–15 min, followed by 2% formaldehyde at 40 mL/min for 10–15 min. The heart, liver, lung (left and right), spleen, kidneys (left and right), and lymph nodes were collected and placed into the 10% formaldehyde for secondary fixation at room temperature. Histological analysis with H&E and Prussian blue staining was conducted for pathological evaluation and MNP retention, respectively, by the Department of Pathology, Chang Gung Memorial Hospital. The image digitalization was conducted by ScanScope^®^ CS System with Aperio Slide Scanner (Major Instrument, Reston, VA, USA) in the pathology core of Chang Gung Molecular Medicine Research Center.

### 2.10. Statistical Analysis

The values are presented as mean ± SE. Statistical analysis was performed with a 2-way analysis of variance (ANOVA) or repeated-measures ANOVA, followed by post hoc comparisons with Duncan’s test (Statistica, StatSoft, Tulsa, OK, USA). A *p*-value < 0.05 was considered statistically significant.

## 3. Results

### 3.1. Enzyme Activity of Immobilized rtPA

**Figure 1:**
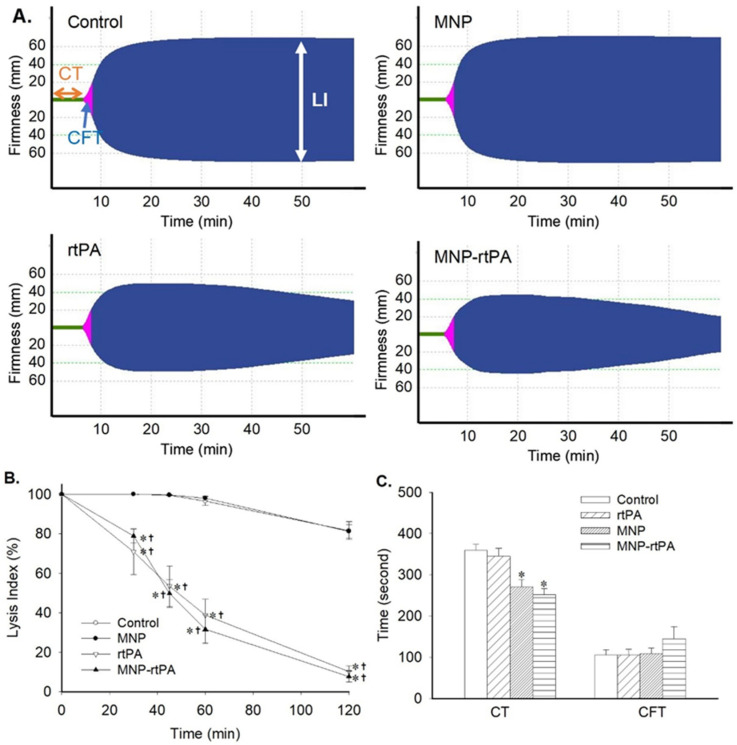
The thrombolytic activity of the immobilized rtPA was preserved after covalent binding to the MNP. (**A**) Representative effects of the rtPA (2 μg/mL), MNP (21 μg/mL), and equivalent MNP-rtPA on thromboelastogram profiles; (**B**) thrombolysis was assessed by % lysis index with time; and (**C**) clotting time (CT) and clot formation time (CFT) were derived from thromboelastograms (*n* = 6). * *p* < 0.05 vs. the control values. † *p* < 0.05 vs. the corresponding MNP values.

The representative thromboelastograms demonstrate that the immobilized rtPA exerted similar enzyme activity as that of the free rtPA, with LI at 60 min (LI_60_) of 33% vs. 49%, respectively, whereas the MNP per se exerted a similar pattern of response in firmness during clot formation as that of the vehicle control (Figure 1A). LI at 120 min (LI_120_) in either the control or MNP group was minimal and averaged 82 ± 5% and 81 ± 4%, respectively, whereas the LI_120_ in the rtPA vs. the MNP-rtPA group was 10 ± 3% and 8 ± 3% (Figure 1B), suggesting that the thrombolytic activity of the immobilized rtPA was similar to that of the free rtPA. However, a significant decrease in the clotting time of the MNP and MNP-rtPA group was observed (Figure 1C), suggesting that coagulation was facilitated in the presence of chitosan-coated MNPs. Nevertheless, there was no significant difference among groups with respect to CFT (Figure 1C); no significant difference was observed in maximal clot firmness or α angle.

**Figure 2:**
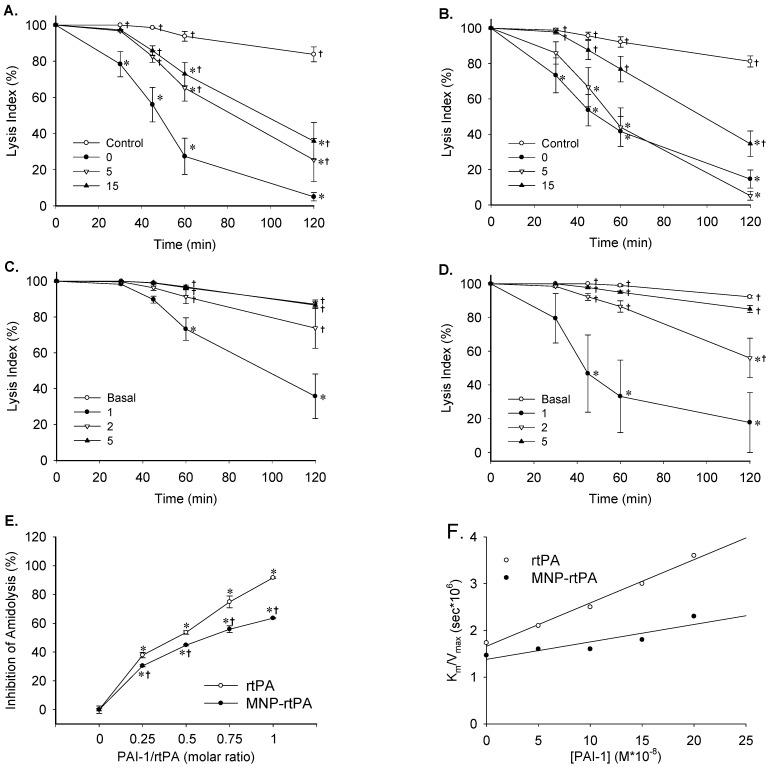
Covalent immobilization on MNP protects the rtPA from inactivation. Thrombolysis induced by free (**A**) and immobilized (**B**) rtPAs (2 μg/mL) after incubation with whole blood at the indicated time (0–15 min) is presented as % lysis index (*n* = 5–9). Blood withdrawal for analysis was conducted 1, 2, and 5 min after i.v. administration of free (**C**) and immobilized (**D**) rtPAs (0.2 mg/kg) (*n* = 4–6). Amidolysis induced by free and immobilized rtPAs as a function of molar ratios (**E**) and PAI-1 concentration (**F**) was determined (*n* = 3). * *p* < 0.05 vs. the control values. † *p* < 0.05 vs. the corresponding 0 min (**A**,**B**), 1 min (**C**,**D**), and rtPA (**E**) values.

The lysis index from thromboelastograms demonstrates that the free rtPA (2 μg/mL) exerted significantly reduced thrombolysis activity within 5 to 15 min of incubation (Figure 2A). In contrast, the thrombolysis activity of the MNP-rtPA remained similar after incubation with whole blood for 5 min (Figure 2B), suggesting a protective effect of immobilization. Nevertheless, a 15-min incubation significantly reduced the LI of the MNP-rtPA (*p* < 0.05). Subsequently, an ex vivo assay was conducted by an analysis of the thrombolysis activity of the free rtPA vs. the immobilized rtPA after circulating in vivo for 1 to 5 min before blood analysis.

With one-min circulation, the free vs. immobilized rtPA exerted an LI_120_ of 36 ± 12% and 18 ± 18%, respectively. With 2-min circulation, the LI_120_ of the free rtPA was not different from that of the basal group (Figure 2C), suggesting most enzymes were inactivated after 2 min in circulation; however, the LI_120_ of the MNP-rtPA remained 56 ± 12% after a 2-min circulation (Figure 2D). The pharmacokinetic results suggest that part of the enzyme activity with the MNP-rtPA was preserved in vivo.

Since the endogenous PAI-1 may account for the significant inhibition of the rtPA activity in the whole blood [2], PAI-1-induced inhibition on the amidolytic activity of the free rtPA vs. the immobilized rtPA was determined. It appears that the inhibitory effects of the PAI-1 were significantly attenuated with the immobilized rtPA in every PAI-1/rtPA ratio studied, suggesting that the enzyme activity of the rtPA was protected from the PAI-1 after immobilization (Figure 2E). Furthermore, the inhibition constant (*K_i_*) was determined by measuring the rtPA-induced hydrolysis of S-2288, an artificial substrate, in the absence or presence of the PAI-1 at various concentrations. The double-reciprocal plots of initial velocities (V) vs. [S-2288] were generated, and then, the slope values (*k_m_*/*V_max_*) were plotted as a function of [PAI-1] (Figure 2F). The *K_i_* values in the rtPA vs. the MNP-rtPA were 0.18 vs. 0.38 μM, respectively, suggesting that rtPA immobilization attenuated the inhibitory effect of the PAI-1.

### 3.2. Magnetic Guiding Effects

**Figure 3:**
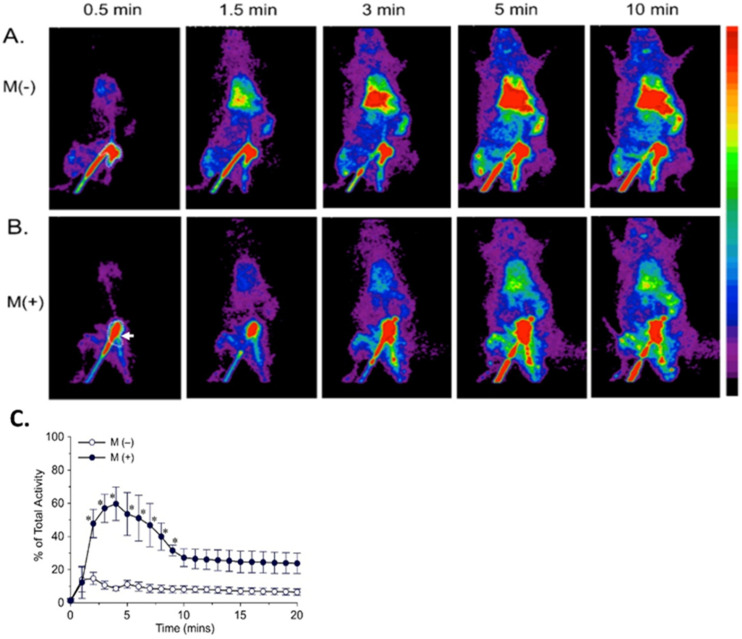
Representative μSPECT images of ^99m^Tc-MNP-rtPA in a rat embolic model. The images demonstrated the biodistribution of ^99m^Tc-MNP-rtPA in the absence (**A**) and presence (**B**) of magneto-guidance (white arrow) in a rat embolic model. The radio activities of the injection site denoted by the dotted lines in (**A**,**B**) were summarized with time in ((**C**); *n* = 3). Dynamic scintigraphic images were collected from 5 min before to 1 h after the administration of ^99m^Tc-MNP-rtPA with 10-sec intervals per image. Each presented image is a fused image of 30 s. * *p* < 0.05 vs. the group without magneto-guidance.

To evaluate the effects of magneto-guidance on the distribution of the MNP-rtPA in vivo, scintigraphic imaging using small animal SPECT was performed in a rat embolic model. Figure 3A,B demonstrates that the ^99m^Tc-MNP-rtPA accumulated mainly in the liver, lung, and spleen, in addition to the injection site. The accumulation of the ^99m^Tc-MNP-rtPA in these organs increased rapidly in minutes after administration and remained elevated in the following hour. The placement of an NdFeB magnet by the left iliac artery appeared to prevent or delay MNP accumulation in these organs. At 3 min after administration, magnet application reduced the radioactivity in the liver/spleen and lung by 56% and 63%, respectively. In the injection area, the magneto-guidance increased radioactivity significantly to a peak of 6.9-fold (Figure 3C), suggesting local MNP retention in response to magnet application.

**Figure 4:**
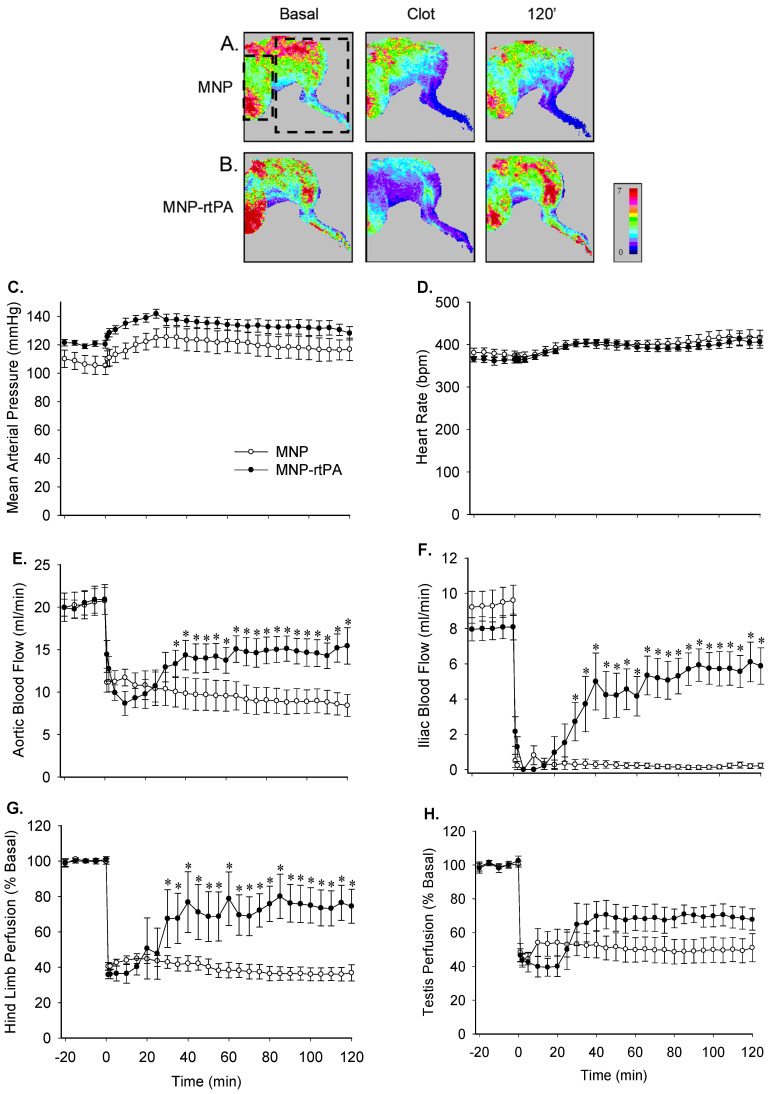
Thrombolytic effects of the MNP-rtPA in a rat embolic model. Representative tissue perfusion of the left hind limb and testis of rats in response to the MNP (**A**) or MNP-rtPA (**B**) targeting was acquired with a laser speckle imager in the designated area, as illustrated with the left and right squares in mean arterial pressure (**C**), heart rate (**D**), aortic blood flow (**E**), iliac blood flow (**F**), hind limb perfusion (**G**), and testis perfusion (**H**), which were measured after introducing a whole blood clot (1.5 × 2 mm) into the left iliac artery at time 0. MNP-rtPA (0.2 mg/kg) or equivalent MNP (2 mg/kg) were administered from the right iliac artery 5 min after introducing the clot with magnetic guiding (*n* = 8). * *p* < 0.05 vs. the MNP values.

Figure 4A,B illustrates representative laser perfusion images of the left hind limb and testis in two rats treated with the MNP or MNP-rtPA, respectively. The introduction of a piece of preformed clot to the left iliac artery immediately reduced tissue perfusion of the left hind limb and testis by 30%-52%. With magnetic guiding, the MNP-rtPA (Figure 4B), but not the MNP (Figure 4A), restored tissue perfusion at 120 min after administration. Figure 4C–H provides the hemodynamic results in response to the MNP vs. the MNP-rtPA. No difference was observed in the mean arterial pressure (Figure 4C), heart rate (Figure 4D), or testis perfusion (Figure 4H) between the treatments of the MNP vs. the MNP-rtPA. In response to clot introduction, the blood flow/perfusion of the abdominal aorta (Figure 4E), iliac artery (Figure 4F), the left hind limb (Figure 4G), and testis were reduced to 52 ± 4%, 0, 40 ± 2%, and 44 ± 3% of the basal levels, respectively (*n* = 16). As early as 25 min after administration of the MNP-rtPA with magnetic guidance, the aortic blood flow, iliac blood flow, and hind limb perfusion were significantly higher than the MNP group and were restored to 73 ± 6%, 74 ± 10%, and 74 ± 10% of the basal levels at the end of the 2-hr observation period, respectively (*n* = 8). The reperfusion time of the iliac blood flow to 50% of the basal level was 45 ± 8 min after the administration of the MNP-rtPA. However, no significant difference was found in the testis perfusion between the MNP- and MNP-rtPA-treated groups.

### 3.3. Toxicology Evaluation

**Figure 5:**
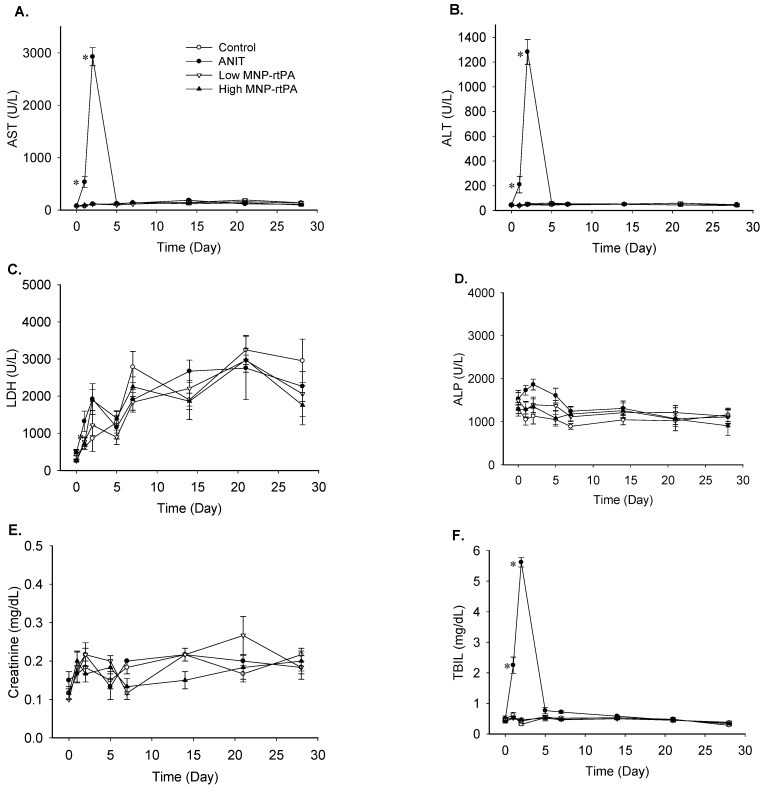
Toxicological assessments in anesthetized rats treated with low and high doses of MNP-rtPA. Blood samples were collected with a permanent catheter in the jugular vein before and after the administration of the vehicle (control), ANIT (α-Naphthylisothiocyanate, 60 mg/kg), and low (0.15 mg/kg) or high (1.5 mg/kg) doses of the immobilized rtPA. The dosages of the MNP in MNP-rtPA were equivalent to 2 vs. 20 mg/kg in low vs. high doses of MNP-rtPA, respectively (*n* = 6). Serum levels of aspartate aminotransferase (AST, (**A**)), alanine aminotransferase (ALT; (**B**)), lactate dehydrogenase (LDH; (**C**)), alkaline phosphatase (ALP; (**D**)), creatinine (**E**), and total bilirubin (TBIL; (**F**)) were determined by a hematologic analyzer. * *p* < 0.05 vs. the control values.

To evaluate the potential toxicity of the MNP-rtPA in vivo, biochemical and hematological analyses were performed on blood withdrawn from the rat at the indicated time after intravenous administration. Figure 5 illustrates that the MNP-rtPA at low or high doses exerted no effect on the serum levels of aspartate aminotransferase (AST), alanine aminotransferase (ALT), lactate dehydrogenase (LDH), alkaline phosphatase (ALP), creatinine, and total bilirubin (TBIL), whereas ANIT, a positive control, significantly increased the ALT, AST, and TBIL levels within 1~2 days after administration. However, the levels returned to normal within five days after injection.

**Figure 6:**
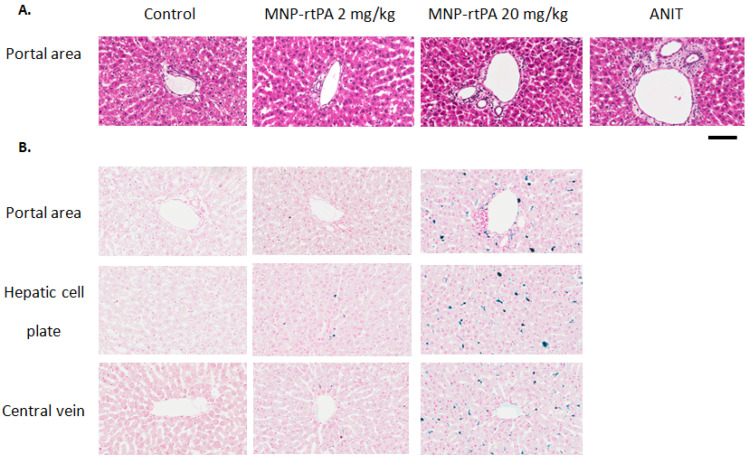
Hepatic retention of the MNP after intravenous injection of MNP-rtPA. H&E stain (**A**) and Prussian blue stain (**B**) were conducted in the liver tissue harvested four weeks after i.v. administration of MNP-rtPA with MNP doses of 2 vs. 20 mg/kg or α-Naphthylisothiocyanate (ANIT, 60 mg/kg). Results are representative of three rats. Scale bar = 100 μm.

A histology analysis of MNP retention was conducted 4 weeks after i.v. administration of the MNP-rtPA. Figure 6A shows the H&E stain of the liver sections. Severe portal edema and neutrophil infiltration were observed in tissues from the rats subjected to ANIT, and to a lesser degree, in rats treated with 20 mg/kg of the MNP-rtPA. In contrast, such an effect was not observed in the groups of the control or MNP-rtPA of 2 mg/kg. In staining for iron using Prussian blue, significant MNP deposition was observed in the portal area, hepatic cell plate, and central vein areas in rats receiving an MNP-rtPA of 20 mg/kg, but much less in the low-dose group (Figure 6B).

## 4. Discussion

**Figure 7:**
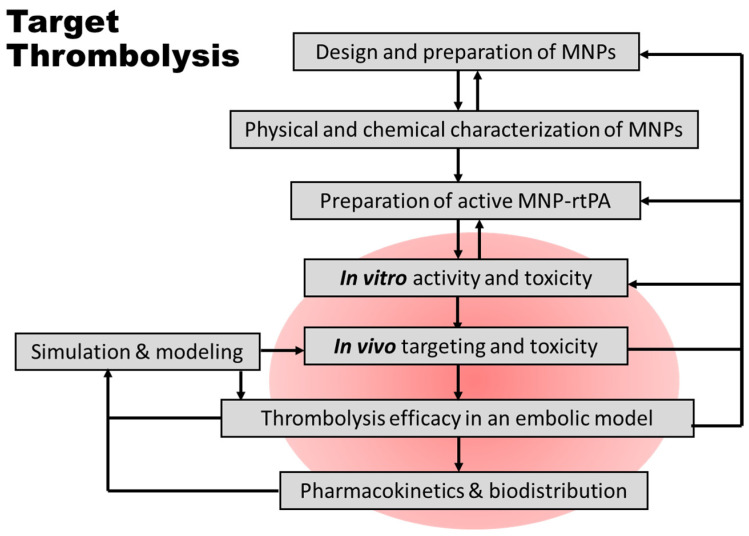
Schematic diagram of the proposed strategies in the development of thrombolytic nanocomposites with magnetic nanoparticles (MNPs). The arrows indicate sequences or feedback considerations in the development for optimization. This translational study focuses on the pharmacological/toxicological evaluation of the rtPA immobilized on chitosan-coated MNPs, as included in the red area. The strategy is modified from the one published previously [6] and may be applied to ligand targeting in addition to magnetic targeting.

This study provides an investigation of the thrombolysis induced by a chitosan-MNP-rtPA in vitro and in vivo, as a critical part of the effort in the development of targeting rtPA nanocomposites (Figure 7). Magnetic guiding significantly enhanced the local retention of the nanocomposite, as shown in this first real-time biodistribution of rtPA nanocomposites in vivo. The immobilization of the rtPA on chitosan-coated MNPs preserved the amydolytic activity of the rtPA from the endogenous inhibitor of PAI-1 and demonstrated a preferable extension of the enzyme activity in vivo. The results are consistent with previous findings on magnetic guiding-induced intravascular retention of MNPs by histology staining [16]. In addition, the rat iliac embolic model appears to be a reproducible functional assessment of targeted thrombolysis; the iliac flow restoration occurred in response to one-fifth of the regular dose of the free rtPA. For the first time, the rtPA nanocomposite did not induce hepatic toxicity at the regular or ten times the dose, despite evident hepatic retention observed in μSPECT images or histological sections.

The enzymatic activity of the chitosan-MNP-rtPA appears to be protected from PAI-1 inhibition, with the *K_i_* value of the PAI-1 on the immobilized rtPA being 2.1-fold greater than that of the soluble rtPA, which may be due to steric hindrance arising from immobilization. Since the PAI-1 inactivates its target proteinases by forming a 1:1 stoichiometric Michaelis complex, followed by constituting salt bridges between the highly positively charged regions of the tPA and three negatively charged amino acids of the PAI-1 [19], we could not rule out possibilities of immobilization-induced conformational change of the rtPA or alteration in the charge-mediated interaction between the rtPA and PAI-1 by chitosan as the coating material. These results are consistent with previous studies that found that the amidolytic activity of the immobilized rtPA may be more resistant to the PAI-1 compared to that of the free rtPA [20]. In addition to magnetic targeting, resistance to the PAI-1 may be attributed to the reduction of the rtPA dose required to achieve thrombolysis efficacy. With magnetic guiding along the iliac artery, re-perfusion of the hind limb, but not the testis, was observed. The results are consistent with previous findings demonstrating that the reperfusion was achieved using rtPA nanocomposites at a lower rtPA dose here and in other thromboembolic models [6].

In the current study, the MNP-rtPA was distributed primarily in the liver, lung, and spleen, which is consistent with previous studies on the biodistribution of MNPs with various coating materials [6], including chitosan [21]. Compared to the administration of the nanocomposites without the magnet, magneto-guidance may retain the MNP-rtPA in the injection site, possibly due to induction of MNP-rtPA aggregation, which may result in reduced organ accumulation during magnetic application. Although previous studies reported a transient increase in ALT, AST, and ALT activities observed after the injection of an oleic acid (OA)-Pluronic-coated MNP [22] or PEG-grafted chitosan/DNA complexes [23], our results suggest no hepatic or hematological toxicity in response to chitosan-coated MNPs with or without the immobilized rtPA.

A regular dose of the MNP-rtPA caused minimal iron retention in all organs studied 28 days after administration, indicating that it is unlikely to induce a concerning level of toxicity. The retention of iron in the liver, lung, and spleen appears to be dose-dependent, with a high capacity for organ retention that is consistent with previous findings of MNPs with various coatings [15]. The retention of the MNP-rtPA in organs is likely due to macrophage uptake [24]. A high dose of the MNP-rtPA (20 mg/kg) was associated with portal area edema, but not elevation of AST and ALT levels, suggesting that elevated liver enzymes in plasma were not a prerequisite for MNP-rtPA-induced portal edema. MNP deposit in the liver appeared to be higher around the portal area (Figure 6B), which is consistent with the blood flow direction. To our knowledge, this is the first report of portal edema induced by MNP-based nanocomposites, which is likely to contribute to an inflammatory response [25,26]. Our results are consistent with previous findings that silica-coated MNP may induce mononuclear infiltration at the portal area of the liver in mice [27]. Although hepatic MNP accumulation may have caused liver toxicity in other studies [17,27,28], a normal portal triad was observed in response to repeated oral administration of MNP in rats [28]. However, the degradation and clearance of the nanocomposite at a higher dose, and its potential long-term toxicity, especially liver, remains to be determined.

In both the chitosan-MNP and chitosan-MNP-rtPA, the CT, but not CFT, was significantly decreased (Figure 1C). Although chitosan was implicated in activating coagulation [29], probably via the enhancement of fibrinogen adsorption [30] and platelet aggregation [31], chitosan-MNP-rtPA still exerted thrombolysis in vivo in the current study. These discrepancies may be due to the different physicochemical properties of chitosan, such as its chemical structure, molecular weight, degree of deacetylation, protonation degree, and whether it is in a liquid or solid state, as well as its interactions with various blood components. However, the thrombolytic activity of the chitosan-MNP-rtPA was comparable to that of rtPA nanocomposites with different coatings [6]. Our results suggest that chitosan nanocomposites may serve as a rtPA carrier in thrombolysis therapy.

## 5. Conclusions

Our study suggests that the immobilized rtPA on the chitosan-MNP given at 20% of a regular dose may effectively achieve targeted thrombolysis and reperfusion in a rat embolic model. Despite portal edema, neutrophil infiltration, and MNP/iron deposit in the liver, lung, and spleen, we did not observe any tissue damage based on the hematology analysis. Our findings provide a comprehensive understanding of the pharmacodynamics and toxicity of a rtPA nanocomposite and may facilitate future applications of targeted thrombolysis with other strategies, such as ligand targeting.

## Data Availability

The authors confirm that the data supporting the findings of this study are available within the article and its Supplementary Materials.

## Supplementary Materials

The following supporting information can be downloaded at: https://www.mdpi.com/article/10.3390/pharmaceutics16050596/s1, Table S1: Hematological measurements in anesthetized rats treated with the MNP or MNP-rtPA.; Figure S1: Concentration-dependent thrombolysis induced by the rtPA; Figure S2: Hematological assessments in anesthetized rats treated with low and high doses of MNP-rtPA; Figure S3: Toxicological assessments in anesthetized rats treated with low and high doses of the MNP; Figure S4: Hematological assessments in anesthetized rats treated with low and high doses of the MNP; Figure S5: Tissue retention of the MNP in the lung and spleen after intravenous injection of the MNP-rtPA.
