# Supplementary material for: Targeted Thrombolysis with Magnetic Nanotherapeutics: A Translational Assessment
Source: Pharmaceutics. 2024 Apr 27;16(5):596. doi: 10.3390/pharmaceutics16050596 (PMC11124959; doi:10.3390/pharmaceutics16050596)
Supplement: Supplementary file 1 [file pharmaceutics-16-00596-s001.zip › pharmaceutics-2960271-supplementary.pdf]

Supplement Materials

Targeted thrombolysis with magnetic nanotherapeutics: A translational assessment

Supplemental Results

The thrombolysis activity of concentrations of free rtPA was first determined. The addition of $CaCl_2$ to the citrated whole blood triggered clot formation, as demonstrated by an increase in elasticity/firmness of the blood samples; co-administration of free rtPA induced thrombolysis with a reduction in the elasticity/firmness of the clot. The thrombolysis effect appears to be concentration-dependent (Fig. S1A). The calculated lysis index (LI) decreased with time, which acted as a function of [rtPA] (Fig. S1B). There was no significant difference in clotting time and clot formation time among these groups (Fig. S1C). The results suggested that thrombolysis induced by rtPA at the concentrations of 0 to 3 mg/mL under experimental conditions may be differentiated and thus determined by TEM.

Suppl. Table 1 summarizes the hematological analysis of blood samples withdrawn from the rats at the end of the observation periods in Fig. 4. No significant difference among the vehicle, MNP, or MNP-rtPA groups was observed.

In toxicological evaluation, biochemical and hematological analysis was performed on blood withdrawn from the rat at the indicated time after intravenous administration. During the 4-week period, MNP-rtPA treatment did not alter the body weight increase compared with that of the control group (Fig. S2A). In addition, the results from the hematological analysis suggested that MNP-rtPA exhibited no significant effects on WBC, RBC, hemoglobin, hematocrit, and platelet. (Fig. S2). In another group of rats, similar results were obtained with intravenous administration of MNP, as illustrated in Figs. S3&S4.

In addition to the liver, MNP/iron deposit was also observed in the lung and spleen for four weeks after i.v. administration of 20 mg/kg of MNP-rtPA, as illustrated in the lower panel of Figs. S5A&B. Prussian blue stain in Fig. S5B also illustrates iron deposit in the spleen in response to MNP-rtPA 2 mg/kg; nevertheless, it is not different from that in the control group. However, no MNP deposit was observed in the lymph node (Fig. S5C) or kidney (Fig. S5D). Other than the liver, there was no evidence suggesting any detrimental effect of even a high dose of MNP-rtPA on the morphology/structure of these organs studied.

Suppl. Table S1. Hematological measurements in anesthetized rats treated with MNP or MNP-rtPA.

Treatments	RBC (10^6/μl)	WBC (10^3/μl)	Platelet (10^3/μl)	HGB (g/dl)	HCT (%)	NE (%)	LY (%)
Vehicle	6.4 ± 0.3	9.5 ± 2.1	899 ± 30	14.2 ± 0.8	44 ± 3	84 ± 4	14 ± 4
MNP	7.0 ± 0.3	7.3 ± 1.4	875 ± 63	14.3 ± 0.3	48 ± 1	70 ± 5	28 ± 5
MNP-rtPA	6.9 ± 0.2	6.1 ± 0.9	889 ± 70	14.6 ± 0.4	49 ± 1	74 ± 3	24 ± 13

Blood samples were collected 110 min after injection of vehicle, rtPA (0.2 mg/kg) covalently bound to MNP (2 mg/kg) or equivalent MNP in the rat embolic model (n=4-6). RBC, red blood cells; WBC, white blood cells; NE, neutrophils; LY, lymphocytes; HGB, hemoglobin; HCT, hematocrit.

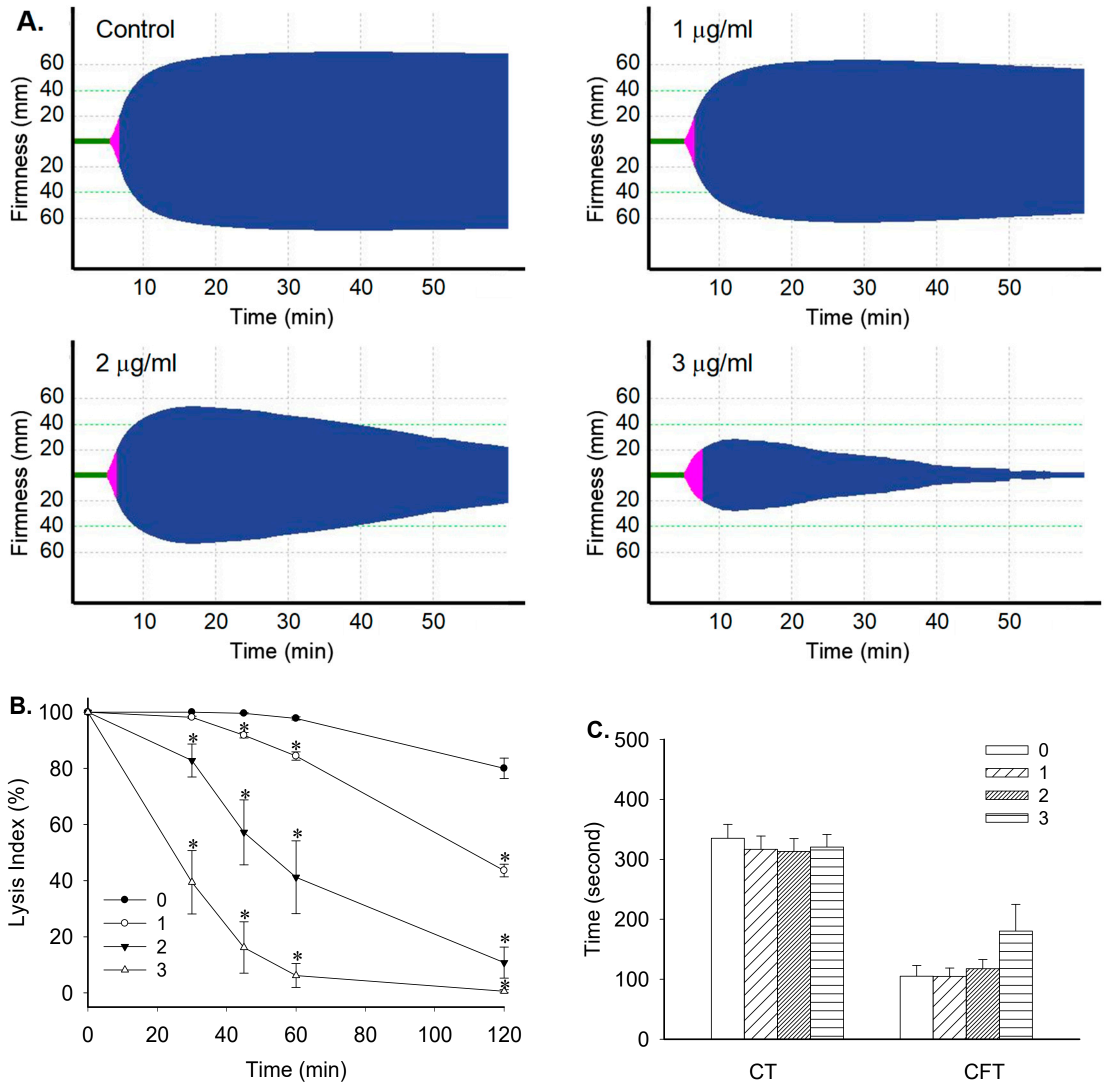

Figure S1. Concentration-dependent thrombolysis induced by rtPA. Representative effects of rtPA on thromboelastogram profiles are illustrated in (A). Concentration-activity relationship of free rtPA at various concentrations was measured by thromboelastometry (B). Clotting time (CT) and clot formation time (CFT) was derived from thromboelastograms as defined by the manufacturer (C). *, $p < 0.05$ compared with the control values.

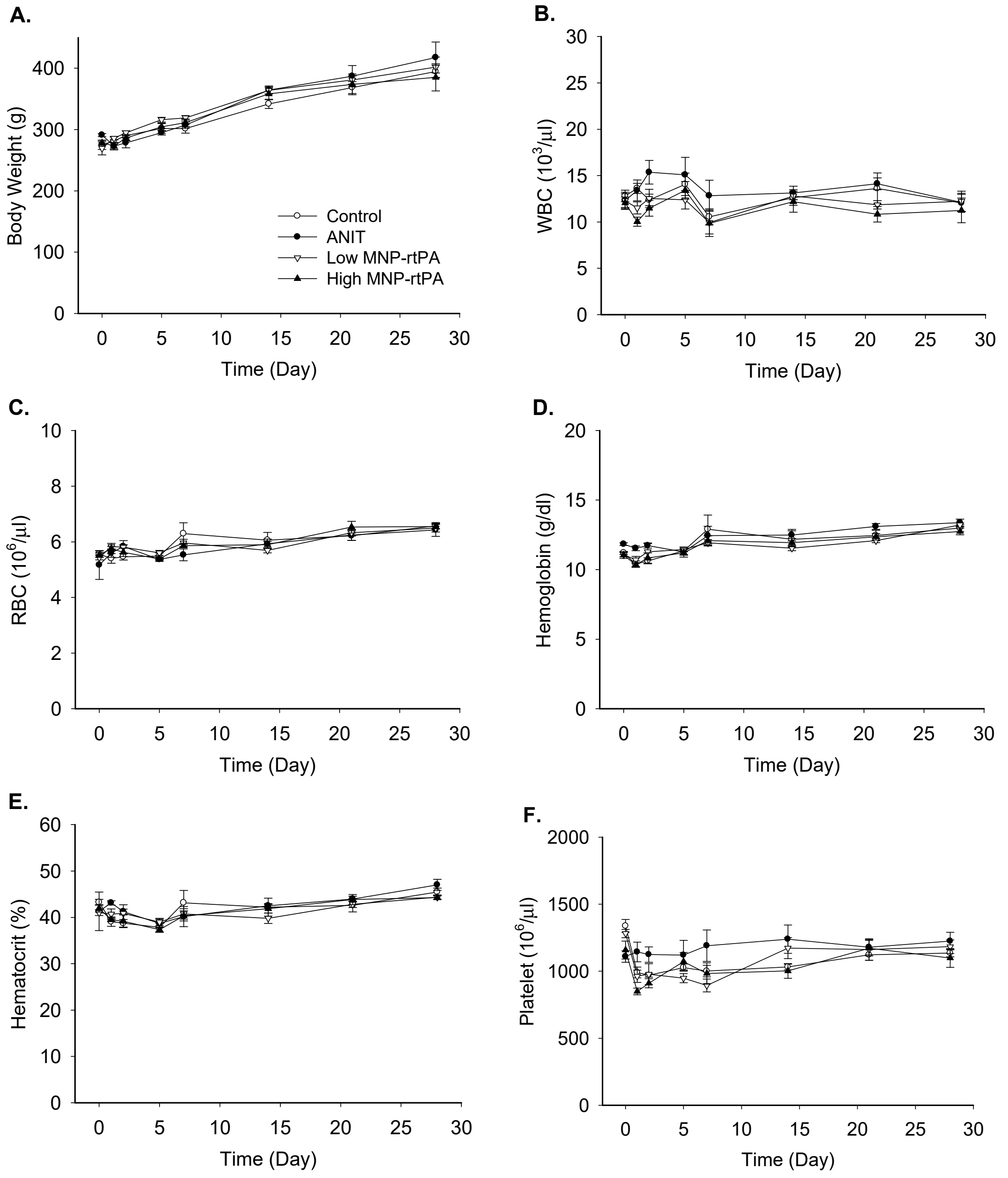

Figure S2. Hematological assessments in anesthetized rats treated with low and high doses of MNP-rtPA. Body weight (A) and hematological parameters (B-F) were measured before and after administration of vehicle (control), ANIT (α-Naphthylisothiocyanate, 60 mg/kg), low (0.15 mg/kg) or high (1.5 mg/kg) doses of immobilized rtPA for four weeks. The dosages of MNP in MNP-rtPA were equivalent to 2 *vs.* 20 mg/kg in low *vs.* high MNP-rtPA, respectively (n=6). Values are presented as mean ± SE. RBC, red blood cells; WBC, white blood cells.

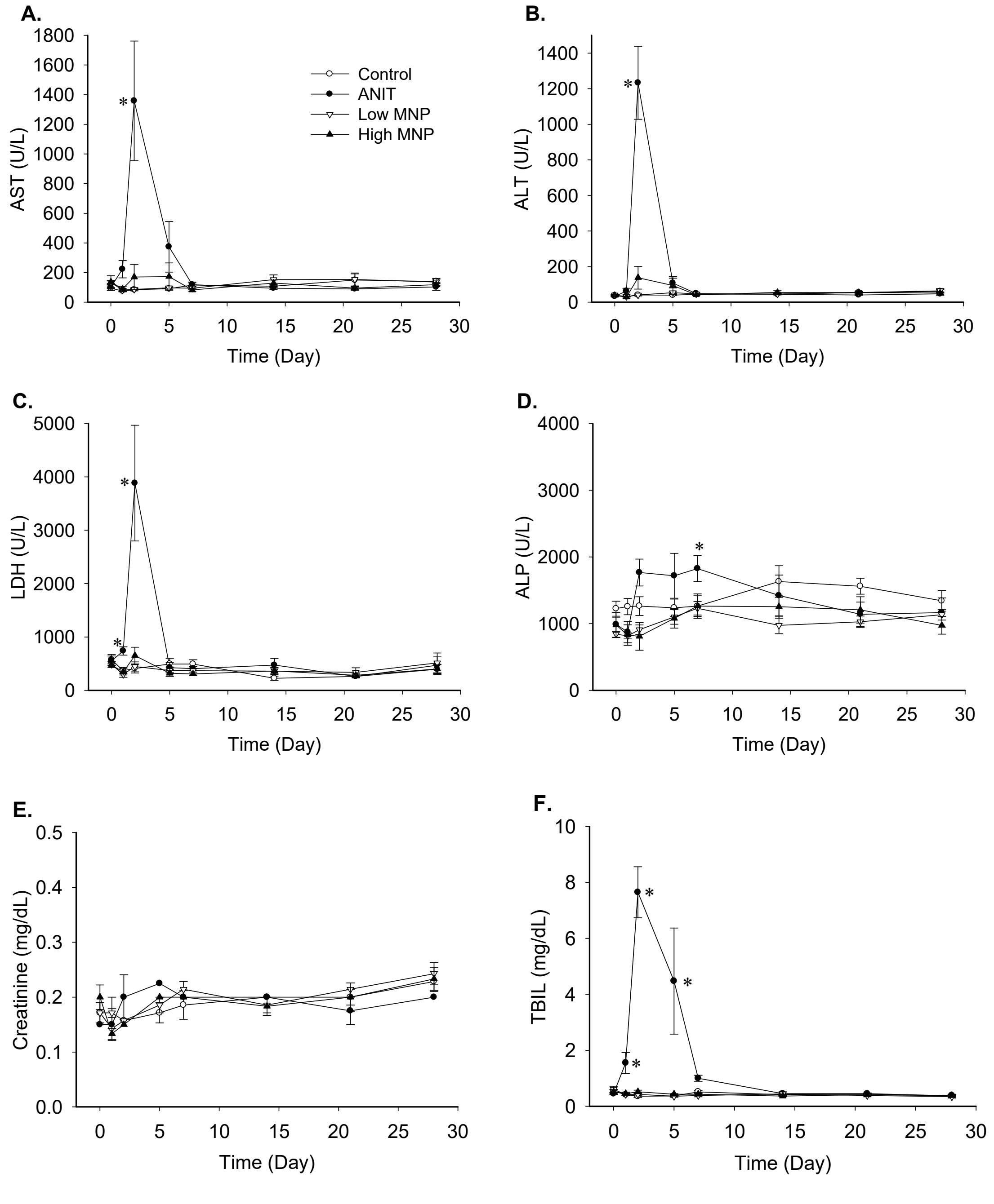

Figure S3. Toxicological assessments in anesthetized rats treated with low and high doses of MNP. Blood samples were collected with a catheter in the jugular vein before and after administration of vehicle (control), ANIT (α-Naphthylisothiocyanate, 60 mg/kg), low (2 mg/kg), or high (20 mg/kg) doses of MNP (n=4-7). Serum levels of aspartate aminotransferase (AST, A), alanine aminotransferase (ALT; B), lactate dehydrogenase (LDH; C), alkaline phosphatase (ALP; D), creatinine (E), and total bilirubin (TBIL; F) were determined by a hematologic analyzer. Values are presented as mean ± SE. *, p<0.05 compared with the control values.

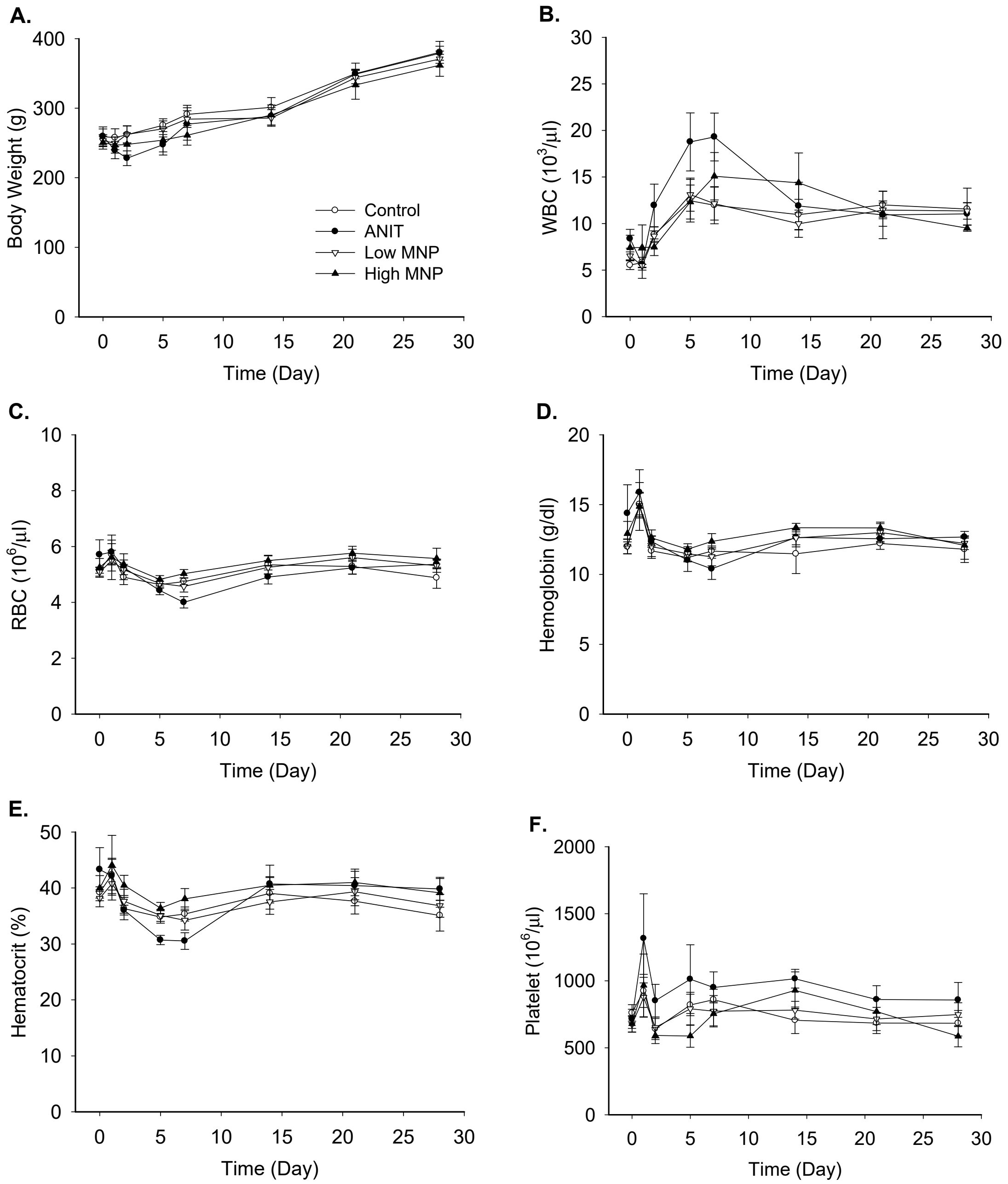

Figure S4. Hematological assessments in anesthetized rats treated with low and high doses of MNP. Body weight (A) and hematological parameters (B-F) were measured before and after administration of vehicle (control), ANIT (α-Naphthylisothiocyanate, 60 mg/kg), low MNP (2 mg/kg), or high MNP (20 mg/kg) (n=4-7)) for four weeks. Values are presented as mean ± SE. RBC, red blood cells; WBC, white blood cells.

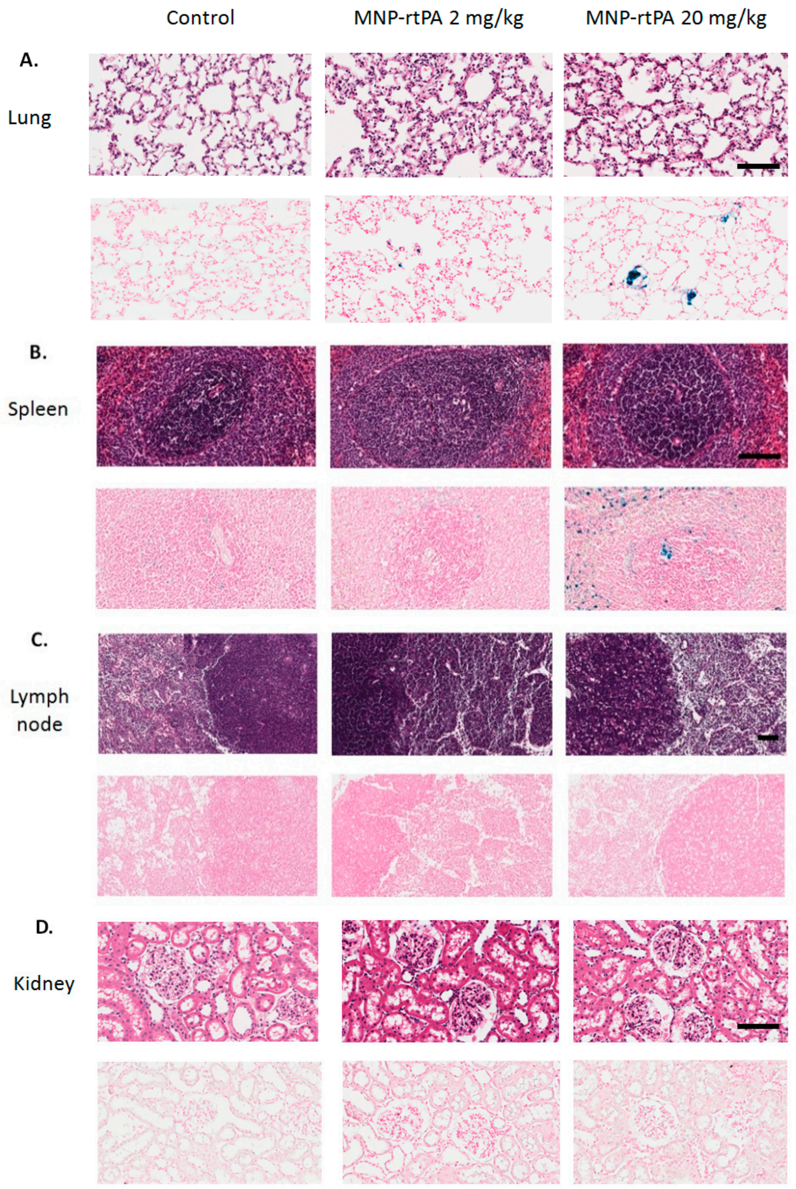

Figure S5. Tissue retention of MNP in the lung and spleen after intravenous injection of MNP-rtPA. H/E stain (A) and Prussian blue stain (B) were conducted in the lung (A), spleen (B), lymph node (C), and kidney (D) harvest four4 weeks after intravenous administration of MNP-rtPA with MNP doses of 2 *vs.* 20 mg/kg. Results are representative of three rats. Scale bar = 100 µm.
